# Spatio-temporal gait parameters obtained from foot-worn inertial sensors are reliable in healthy adults in single- and dual-task conditions

**DOI:** 10.1038/s41598-021-88794-4

**Published:** 2021-05-13

**Authors:** J. Soulard, J. Vaillant, R. Balaguier, N. Vuillerme

**Affiliations:** 1 University of Grenoble Alpes, AGEIS, Grenoble, France; 2 Grenoble Alpes University Hospital, Grenoble, France; 3 LabCom Telecom4Health, University Grenoble Apes and Orange Labs, Grenoble, France; 4 Institut Universitaire de France, Paris, France

**Keywords:** Medical research, Signs and symptoms

## Abstract

Inertial measurement units (IMUs) are increasingly popular and may be usable in clinical routine to assess gait. However, assessing their intra-session reliability is crucial and has not been tested with foot-worn sensors in healthy participants. The aim of this study was to assess the intra-session reliability of foot-worn IMUs for measuring gait parameters in healthy adults. Twenty healthy participants were enrolled in the study and performed the 10-m walk test in single- and dual-task ('carrying a full cup of water') conditions, three trials per condition. IMUs were used to assess spatiotemporal gait parameters, gait symmetry parameters (symmetry index (SI) and symmetry ratio (SR)), and dual task effects parameters. The relative and the absolute reliability were calculated for each gait parameter. Results showed that spatiotemporal gait parameters measured with foot-worn inertial sensors were reliable; symmetry gait parameters relative reliability was low, and SR showed better absolute reliability than SI; dual task effects were poorly reliable, and taking the mean of the second and the third trials was the most reliable. Foot-worn IMUs are reliable to assess spatiotemporal and symmetry ratio gait parameters but symmetry index and DTE gait parameters reliabilities were low and need to be interpreted with cautious by clinicians and researchers.

## Introduction

Locomotion is an acquired fundamental human motor activity^1^. Motor command allows muscle contraction control and subsequent joint movements, permitted by the integration of multiple sensory information continuously^2^. Optical motion analysis systems are considered as the gold-standard to assess gait parameters^3^. These parameters allow the understanding of the process of human locomotion^3^. However, their clinical use is poor and “gait testing” is still considered as “research” in the United states^4^. Indeed, these systems are expensive, they further require experienced individuals for data collection and analysis and are difficult to transport between care units^4^. On the contrary, clinicians are looking for easy to use, transportable and affordable gait analysis systems. Inertial measurement units (IMUs) containing 3-axis accelerometers fully meet these specific needs, making them increasingly popular and usable in clinical routine, to diagnose, determine severity of disease, select appropriate treatment and predict prognosis^5^. IMUs can be placed for instance on trunk, sternum, shank or feet^6^. Foot-worn IMUs allow quantification of symmetry between left and right foot^7^ (i.e. to assess differences in the contribution of each limb to propulsion and control during walking^8,9^) and permit to differentiate the phase of stance in load, foot flat and push ratio^10^. However, to allow in clinical practice the expanded use of these sensors, testing their reliability is crucial. When a measurement is repeated by the same participant, the reliability refers to the reproducibility of this measurement^11^. More-precisely, the intra-session reliability refers to the reliability of different trials performed during the same session of time^11,12^. The reliability should always be reported by authors to better describe the sources of imprecision in clinical trials results^11^.

A very recent meta-analysis questioning validity and reliability of wearable inertial sensors in healthy adults during walking^3^ have reported seven published studies that have used foot-worn sensors to assess spatiotemporal gait parameters^10,13–18^, among which two of them (28.6%) used Physilog IMUs^10,17^. Concurrent validity of the Physilog foot-worn inertial sensors and associated gait analysis package for the spatiotemporal gait analysis has previously been validated as compared to optical motion capture system in healthy adults^17,19^ but also in pathological populations (i.e., children with cerebral palsy^20^, patients with Parkinson disease^21^ or after a stroke^22^). However, to the best of our knowledge, no study has assessed the intra-session reliability of spatiotemporal gait parameters or the number of trial required to obtain reliable gait parameters. Since the concurrent validity between Physilog foot-worn inertial sensors and associated gait analysis package and optical motion capture systems has already been established^17,19^, the present study was designed to solely focus on intra-session reliability of Physilog foot-worn inertial sensors for measuring gait parameters in healthy adults. Besides, the meta-analysis of Kobsar et al. focused on the assessment of spatiotemporal gait parameters measured in single-task condition^3^. However, the assessment of gait in dual-task condition is recommended for ecological validity to assess safety risk and function progression^23^. Indeed, the ability to maintain balance while simultaneously performing a cognitive task is essential for daily living and has been implicated as a risk factor of falls in older adults^24,25^. Dual task is defined as “the concurrent performance of two tasks that can be performed independently and have distinct and separate goals”^23^. The dual task effect can further be calculated to quantify costs of multitasking^23,26^. Interestingly, when comparing daily-living and in-lab gait pattern in older adults, Hillel et al. in 2019 have recently reported that gait parameters “obtained during daily-living were closer in value to the dual task values measured in the laboratory setting” (^27^, page 1). Furthermore, gait characteristics in dual task allowed better classification of patients with and without cognitive impairment than single task^28^.

Besides, previous studies that have used foot-worn inertial sensors from Physilog to assess spatiotemporal gait parameters during walking at self-selected comfortable speed revealed a high heterogeneity regarding the walking protocols used (e.g. 5 m walking test^17,19^ *versus* 50 m walking test^10^). The 10-m walk test is a clinical test timing how long it takes a patient to walk 10 meters^29^. It was validated in a wide range of conditions and is generalizable to clinical practice even if clear and standardized instructions have to be given to allow accurate data during walking and appropriate clinical decisions^29^. A rolling start and finish was considered as easier and more convenient for patient with neurological conditions^29^. Besides, a self-selected or comfortable speed should be used as less random measurement errors than walking at maximum speed were found^30^. In its original form, the 10-m walk test was based on the measurement of the walking speed (m/s) only, calculated from the time to walk 10 m. Interestingly, the use of IMUs during the performance of this clinical test now allows to further compute relevant data such as spatiotemporal gait parameters and their symmetry.

Along these lines, the aim of the present study was to assess the intra-session reliability of foot-worn Physilog IMUs for measuring gait parameters under single-task and dual-task walking in healthy adult during the 10-m walk test. More precisely, the objectives are 1) to evaluate the reliability, standard error of measurement (SEM) and minimal detectable change (MDC) values of spatiotemporal gait parameters and symmetry gait parameters obtained, (2) to evaluate the reliability, SEM and MDC values of dual task effects obtained from gait parameters, and (3) to determine the number of trials required to ensure reliable gait assessment.

## Methods

### Participants

All participants signed informed consent and approved to participate in Folomi study as healthy control participants^31^. The study was approved by local ethic committee (CPP Ile De France 1, RCB: 2017-A03468-45, date of agreement: July 17th, 2018, Last version: V6.0, June 17th, 2020), is registered in Clinical trials (NCT03761212) and was performed in accordance with relevant guidelines and regulations.

In line with previous studies on reliability on healthy^10,17,19^ and pathological gait^20,22,32,33^, twenty healthy participants (10 first men and 10 first women of healthy participants of the Folomi study^34^) were included in the present study.

Participants should be aged between 18 to 65 years, able to walk 180 m without technical help, with a public health insurance (French social security). The non-inclusion criteria were: (1) Musculo-skeletal, cardio-respiratory or neurologic disease that could affect gait, (2) Hip or knee arthroplasty done, (3) Not able to speak French, (4) Desire of pregnancy in the following 18 months and (5) Adults protected by laws (Article L1121-5)^31^. Demographic and anthropometric characteristics of study participants (age, height, leg height, body mass, body mass index) were obtained from the participants. Leg length (in meters) was measured from the anterior superior iliac spine to the medial malleolus^35^.

### Experimental protocol

A 10-m Walk test (10MWT) was performed on a 14 m walkway for a rolling start (2 m) and finish (2 m) of the 10 m walk^29^, at comfortable walking speed^36^. This walking task was performed in single- and dual-task conditions (3 trials per condition)^31^.

In the dual-task condition, participants had to carry a full cup of water in their dominant hand with the following instruction: “perform both tasks as well as possible”^26,37^. The examiner noted whether there was any spillage of water^26^. This manual task has been shown to significantly alter spatiotemporal gait parameters during walking in healthy adults with significant reduction of gait speed, stride length and cadence^38^.

### Gait assessment

Two wireless inertial sensor systems (Physilog5, 200 Hz, BioAGM, Gait Up, CH), fixed on the dorsal part of the participant’s feet using Velcro straps (behind the base of the fifth metatarsal^22^), were used to collect gait data (Supplementary Fig. S1). Gait assessments were performed by the same examiner.

Spatiotemporal gait parameters (Table 1) were calculated with the Gait Analysis Software provided by Gait Up (CH)^39^. The two first and last steps were removed from the analysis^40,41^ and at least 16 steps were included in the analysis. The mean of left and right gait parameters were calculated and used for calculation of mean gait parameters:

- Speed (m s^−1^): Mean walking stride velocity of forward walking
- Cadence (step/minute): Number of steps in a minute
- Stride length (m): Distance between two consecutive footprints on the ground, from the heel of a foot to the heel of the same foot, one cycle after
- Swing (%): Portion of the cycle during which the foot is in the air and does not touch the ground
- Stance (%): Portion of the cycle during which part of the foot touches the ground
- Double support (%): Portion of the cycle where both feet touch the ground
- Load (%): Portion of the stance between the heel strike and the foot being flat on the ground
- Foot Flat (%): Portion of the stance where the foot is fully flat on the ground
- Push (%): Portion of the stance between the foot being flat on the ground and the toe leaving the ground at take off

Speed, cadence and stride length were normalized to have non dimensional values using the formula described by Hof^42^ and Pinzone et al.^35^ :

$Normalized speed \left( {speed N} \right) = \frac{Speed}{{\sqrt {gl_{0} } }}$ with g the acceleration of gravity (= 9.81 m/s^2^) and l_0_ leg length; $Normalized cadence \left( {cadence N} \right) = Cadence*\sqrt {\frac{{l_{0} }}{g}}$ with g the acceleration of gravity and l_0_ leg length; $Normalized stride length \left( {stride length N} \right) = \frac{stride length}{{l_{0} }}$ with l_0_ leg length.

### Gait symmetry measures

To assess the contribution of each limb to propulsion and control during walking^8,9^, symmetry gait parameters can be used^9^. Among these parameters, the symmetry index (SI)^9^ and the symmetry ratio (SR)^43^ are the more commonly used. The SI, which is the most commonly used and cited in published studies on gait symmetry^9,43,44^, was calculated. SI was calculated with the following formula for each spatiotemporal gait parameter (except double support)^9^:

$$Symmetry Index \left( {SI} \right) = \frac{{\left| { X_{L} - X_{R} } \right|}}{{0.5 \times \left( {X_{L} + X_{R} } \right)}} \times 100\%$$

with X_L_ the value of each parameter of left foot and X_R_ the value of each parameter of right foot.

A value of SI of 0 indicates full symmetry, while SI ≥ 100% indicates its asymmetry.

The symmetry ratio (SR), described as easier to interpret, is recommended on the basis of potential clinical utility in patients after stroke which often display asymmetry between left and right leg^43^. SR was calculated with the following formula for each spatiotemporal gait parameter (except double support)^43^:

$$Symmetry ratio \left( {SR} \right) = \frac{{X_{R} }}{{X_{L} }}$$

with X_L_ the value of each parameter of left foot and X_R_ the value of each parameter of right foot.

A SR of 1 indicates full symmetry, while a SR of 2.0 on gait speed indicates that the right foot is twice faster than left foot.

### Dual task effects parameters

Dual task effects (DTE) were calculated to assess the influence of the addition of a secondary attention demanding task^23,26^. DTE can be calculated as a relative measure of change (DTE) or in percentage (DTE%)^26^. DTE, as the relative measure of change in performance ^45^, was calculated from mean parameters as^26^:

$$DTE = {\text{single task performance}} - {\text{dual task performance }}$$.

DTE has the same unit as the gait parameter that is used in the calculation. A negative value of DTE for gait speed, cadence, stride length, swing and foot flat is associated to better performance in dual task; while a negative value for stance, double support, load and push is associated to worse performance in dual task^26^. DTE can also be calculated as a percentage (DTE%), being unit-less, and permitting comparison between parameters^26^:

$$DTE \% = { }\frac{{{\text{single task performance}} - {\text{dual task performance}}}}{{\text{single task performance}}}X100$$.

Same interpretation as DTE can be done regarding positive and negative values.

### Statistical analysis

Spatiotemporal gait parameters, gait symmetry measures, and dual task effects, were normally distributed (Shapiro–Wilk normality test). A repeated measures analysis of variance (RM-ANOVA) with the number of trials used as within subject factor was performed on gait parameters, ICC and SEM values^46^. When significant effect of the number of trials was found, a Tukey post-hoc test was used to compare differences between trials^46^.

Intraclass correlation coefficients (ICC) were used to compute the relative reliability, and standard error of measurement (SEM) and minimal detectable change (MDC) were used to compute the absolute reliability across the trials 1–2–3 in each condition (single- and dual-task).

The relative reliability was evaluated by the calculation of a 2-way fixed ICC_2,1_ (for absolute agreement)^47^. ICC values inferior to 0 were considered as poor reliability, between 0.01 to 0.2 as slight, between 0.21–0.4 as fair, between 0.41–0.6 as moderate, 0.61–0.8 as substantial and 0.8–1.00 as almost perfect^48^. SEM, expressed in the same unit as gait parameters, corresponds to the absolute measure of the variability of the errors of measurements. SEM informs on the precision of gait parameters of individual examinees^49^. SEM was calculated with the following formula^50^:

$$SEM = SD\sqrt {1 - ICC}$$

with SD the standard deviation of the parameters from all patients and ICC the relative reliability.

MDC is the minimum value for which a difference can be considered as “real”, and was generated with the formula^15^:

$$MDC = SEM \times 1.96 \times \surd 2$$

with SEM the Standard Error of Measurement.

SEM% and MDC% were also calculated as a percentage of mean for each parameter. SEM% values were classified as ‘low’ (SEM% ≤ 10%) or ‘high’ (SEM% > 10%)^51^. MDC% values were classified as ‘low’ (MDC% ≤ 20%), ‘acceptable’ (20% < MDC% < 40%) and ‘high’ (MDC% ≥ 40%)^51^ with a MDC% < 10% considered as excellent and a MDC% < 40% as acceptable^52^. These two classifications were based on previous publications^32,51,53^ as no clear criteria for interpretation of SEM% and MDC% are available.

Limits of agreements (LOA) and Bland and Altman plots of the differences between trials against their mean, were used to assess the magnitude of disagreement between trials. A difference between trials outside the LOA can be considered as real change (Fig. 2 and Supplementary Fig. S2)^53^.

### Ethics approval and consent to participate

The study was approved by local ethic committee (CPP Ile De France 1, RCB: 2017-A03468-45, date of agreement: July 17th, 2018, Last version: V6.0, June 17th, 2020). The study is registered on ClinicalTrials.gov, with the following ID: NCT03761212 and follow the SPIRIT checklist. Written informed consent were obtained from all participants by a physiotherapist or a doctor. Any modification to the initial protocol will be presented to the local ethic committee and has to be accepted before application and registered in ClinicalTrials.gov.

## Results

Healthy adults included in the present study were 44.9 (11.7) years old. They were 174.7 (8.1) meters height and 69.3 (11.6) kg weight.

### Intra-session relative and absolute reliability of gait parameters

Table 1 reports means and standard deviations of gait parameters obtained for each trial and for the three trials, in the single-task and the dual-task condition, and for dual task effects (DTE) and DTE% parameters. *p* values obtained from RM-ANOVA for differences between trials are also reported. Results showed that no significant difference was found between trials in all gait parameters in single- and dual-task conditions and for DTE and DTE% parameters (Table 1).

**Table 1:** Means and standard deviations of gait parameters for each trial and for the three trials, in the single- and dual-task conditions, and DTE and DTE% parameters, with *p* values obtained from RM-ANOVA to compare trials 1,2 and 3.

	Trial 1 Mean (SD)	Trial 2 Mean (SD)	Trial 3 Mean (SD)	Trial 123 Mean (SD)	F	*p* value	Trial 1 Mean (SD)	Trial 2 Mean (SD)	Trial 3 Mean (SD)	Trial 123 Mean (SD)	F	*p* value
	**SINGLE TASK**						**DUAL TASK**					
Spatiotemporal gait parameters												
Speed	1.45 (0.16)	1.49 (0.16)	1.52 (0.16)	1.49 (0.16)	1.04	0.36	1.40 (0.14)	1.45 (0.17)	1.45 (0.15)	1.43 (0.15)	0.54	0.58
Cadence	112.81 (5.66)	114.13 (7.53)	115.62 (5.93)	114.19 (6.42)	0.96	0.39	113.6 (4.89)	114.63 (4.8)	114.66 (5.57)	114.3 (5.04)	0.28	0.75
Slength	1.52 (0.15)	1.54 (0.14)	1.56 (0.15)	1.54 (0.14)	0.35	0.71	1.46 (0.14)	1.50 (0.16)	1.49 (0.14)	1.48 (0.15)	0.29	0.75
DS	20.14 (3.86)	19.12 (3.7)	19.39 (3.97)	19.55 (3.81)	0.38	0.68	20.61 (3.81)	19.69 (3.65)	19.58 (3.71)	19.96 (3.69)	0.46	0.63
Swing	39.72 (1.93)	39.85 (2.16)	40.05 (2.11)	39.87 (2.04)	0.12	0.88	39.37 (1.99)	39.82 (1.94)	39.86 (1.92)	39.69 (1.93)	0.40	0.67
Stance	60.28 (1.93)	60.15 (2.16)	59.95 (2.11)	60.13 (2.04)	0.12	0.88	60.63 (1.99)	60.18 (1.94)	60.14 (1.92)	60.31 (1.93)	0.40	0.67
LDr	12.38 (3.03)	12.49 (2.7)	12.90 (2.85)	12.59 (2.82)	0.18	0.83	12.85 (2.48)	12.94 (2.71)	13.12 (2.88)	12.97 (2.65)	0.05	0.95
FFr	52.13 (4.55)	51.86 (5.01)	51.09 (4.78)	51.69 (4.72)	0.26	0.77	51.40 (4.46)	51.28 (4.89)	50.89 (4.97)	51.19 (4.70)	0.06	0.94
Pur	35.49 (3.62)	35.65 (4.86)	36.01 (3.98)	35.72 (4.12)	0.08	0.92	35.75 (3.77)	35.78 (4.31)	35.99 (4.50)	35.84 (4.13)	0.02	0.98
Normalized spatiotemporal gait parameters												
Speed N	0.48 (0.05)	0.49 (0.05)	0.50 (0.05)	0.49 (0.05)	1.07	0.35	0.46 (0.04)	0.48 (0.05)	0.48 (0.04)	0.47 (0.04)	0.73	0.49
Cadence N	34.80 (1.34)	35.21 (2.00)	35.68 (1.52)	35.23 (1.66)	1.41	0.25	35.06 (1.51)	35.39 (1.54)	35.39 (1.58)	35.28 (1.53)	0.29	0.75
Slength N	1.63 (0.13)	1.65 (0.13)	1.67 (0.13)	1.65 (0.13)	0.50	0.61	1.56 (0.09)	1.59 (0.11)	1.59 (0.1)	1.58 (0.10)	0.67	0.51
Symmetry index gait parameters												
Speed SI	1.81 (1.44)	2.79 (3)	3.05 (2.53)	2.55 (2.43)	1.49	0.24	3.62 (2.88)	3.46 (2.97)	3.14 (2.92)	3.41 (2.88)	0.14	0.87
Cadence SI	0.83 (0.53)	1.39 (2.89)	1.14 (2.03)	1.12 (2.04)	0.36	0.70	1.4 (2.77)	1.28 (1.4)	1.6 (3.18)	1.43 (2.53)	0.08	0.92
Slength SI	2.24 (1.67)	2.55 (2.05)	2.68 (1.81)	2.49 (1.83)	0.30	0.74	3.77 (2.44)	3.47 (2.39)	3.46 (2.52)	3.56 (2.41)	0.10	0.90
Swing SI	3.58 (3.23)	3.08 (2.76)	3.78 (3.28)	3.48 (3.06)	0.27	0.76	4.22 (3.60)	3.99 (3.37)	4.31 (4.21)	4.17 (3.68)	0.04	0.96
Stance SI	2.35 (2.16)	2.03 (1.79)	2.51 (2.13)	2.30 (2.01)	0.29	0.75	2.74 (2.43)	2.64 (2.34)	2.82 (2.76)	2.73 (2.47)	0.02	0.98
LDr SI	11.77 (7.69)	14.12 (9.16)	12.49 (10.4)	12.8 (9.05)	0.35	0.71	11.34 (8.86)	11.72 (8.62)	11.77 (8.28)	11.61 (8.45)	0.01	0.99
FFr SI	4.24 (2.97)	5.39 (4.98)	4.87 (3.86)	4.83 (3.98)	0.40	0.67	5.19 (4.41)	4.86 (3.61)	5.63 (5.23)	5.23 (4.40)	0.15	0.86
Pur SI	5.54 (3.44)	6.88 (5.22)	6.94 (5.27)	6.45 (4.69)	0.56	0.57	8.14 (8.11)	7.70 (6.62)	8.44 (7.44)	8.09 (7.29)	0.05	0.95
Symmetry ratio gait parameters												
Speed SR	1.01 (0.02)	1.03 (0.03)	1.03 (0.03)	1.02 (0.03)	1.41	0.25	1.04 (0.03)	1.02 (0.04)	1.03 (0.03)	1.03 (0.04)	1.41	0.25
Cadence SR	0.99 (0.01)	1.00 (0.03)	1.00 (0.02)	1.00 (0.02)	0.82	0.44	1.01 (0.03)	0.99 (0.02)	1.00 (0.04)	1.00 (0.03)	0.75	0.48
Slength SR	1.02 (0.02)	1.03 (0.02)	1.03 (0.02)	1.02 (0.02)	0.53	0.59	1.04 (0.03)	1.02 (0.04)	1.04 (0.03)	1.03 (0.03)	1.56	0.22
Swing SR	0.98 (0.04)	0.99 (0.04)	1.00 (0.05)	0.99 (0.04)	1.23	0.30	0.99 (0.05)	0.98 (0.05)	0.99 (0.06)	0.99 (0.05)	0.22	0.80
Stance SR	1.02 (0.03)	1.01 (0.03)	1.00 (0.03)	1.01 (0.03)	1.21	0.31	1.01 (0.04)	1.02 (0.03)	1.01 (0.04)	1.01 (0.04)	0.14	0.87
LDr SR	1.05 (0.14)	1.06 (0.17)	1.04 (0.18)	1.05 (0.16)	0.06	0.94	1.03 (0.16)	1.02 (0.16)	1.04 (0.15)	1.03 (0.15)	0.11	0.89
FFr SR	0.98 (0.05)	0.98 (0.07)	0.98 (0.06)	0.98 (0.06)	0.02	0.98	0.96 (0.05)	0.97 (0.05)	0.96 (0.06)	0.97 (0.06)	0.32	0.73
Pur SR	1.02 (0.06)	1.03 (0.09)	1.02 (0.09)	1.02 (0.08)	0.05	0.95	1.06 (0.12)	1.04 (0.10)	1.05 (0.11)	1.05 (0.11)	0.15	0.86
	**DTE PARAMETERS**						**DTE% PARAMETERS**					
Speed	0.05 (0.14)	0.04 (0.14)	0.08 (0.13)	0.06 (0.14)	0.36	0.70	2.64 (10.17)	2.58 (9.36)	4.73 (8.04)	3.32 (9.13)	0.35	0.71
Cadence	− 0.79 (4.65)	− 0.51 (5.55)	0.97 (4.32)	− 0.11 (4.84)	0.75	0.48	− 0.80 (4.11)	− 0.70 (5.31)	0.77 (3.62)	− 0.25 (4.39)	0.79	0.46
Slength	0.06 (0.10)	0.05 (0.10)	0.07 (0.09)	0.06 (0.09)	0.25	0.78	3.64 (6.51)	2.97 (6.46)	4.21 (5.58)	3.61 (6.12)	0.20	0.82
DS	− 0.46 (2.39)	− 0.56 (2.23)	− 0.19 (1.75)	− 0.41 (2.11)	0.16	0.85	− 3.05 (11.35)	− 3.75 (11.81)	− 1.89 (10.00)	− 2.9 (10.92)	0.14	0.87
Swing	0.36 (1.24)	0.02 (1.64)	0.18 (1.34)	0.19 (1.4)	0.27	0.76	0.86 (3.16)	− 0.05 (4.36)	0.38 (3.33)	0.40 (3.62)	0.31	0.74
Stance	− 0.36 (1.24)	− 0.02 (1.64)	− 0.18 (1.34)	− 0.19 (1.4)	0.27	0.76	− 0.61 (2.03)	− 0.09 (2.64)	− 0.34 (2.26)	− 0.34 (2.30)	0.25	0.78
LDr	− 0.47 (1.27)	− 0.45 (1.16)	− 0.22 (0.91)	− 0.38 (1.11)	0.32	0.73	− 5.37 (10.82)	− 4.12 (9.94)	− 1.94 (7.64)	− 3.81 (9.51)	0.66	0.52
FFr	0.73 (2.22)	0.58 (3.26)	0.20 (2.11)	0.50 (2.55)	0.23	0.80	1.31 (4.24)	0.91 (6.01)	0.33 (4.25)	0.85 (4.84)	0.20	0.82
Pur	− 0.26 (1.61)	− 0.13 (2.59)	0.02 (1.72)	− 0.12 (1.99)	0.10	0.91	− 0.77 (4.44)	− 0.81 (8.01)	0.12 (4.61)	− 0.49 (5.84)	0.16	0.85

Abbreviations: DS = double support, N = normalized, slength = stride length, LDr = Load Ratio, FFr = Foot flat ratio, Pur = Push ratio, SI = symmetry index, SR = symmetry ratio.

Tables 2 and 3 report relative reliability with intraclass correlation coefficients (ICC) and limits of agreement (LOA) values for spatiotemporal gait parameters obtained in the single-task and the dual-task condition, respectively.

**Table 2:** Intraclass correlation coefficients (ICC) and limits of agreement (LOA) values for spatiotemporal gait parameters obtained in single task condition between the mean of the first and second trials (T1-T2), the first and the third trials (T1-T3), the second and the third trials (T2-T3) and the means of the three consecutive trials (T1-T2-T3).

	ICC 12 (lb–ub)	LOA (lb–ub)	ICC 13 (lb–ub)	LOA (lb–ub)	ICC 23 (lb–ub)	LOA (lb–ub)	ICC(123)
**SINGLE TASK**							
*Spatiotemporal gait parameters*							
Speed	0.91 (0.73–0.96)	(− 0.15–0.07)	0.84 (0.30–0.94)	(− 0.20–0.05)	0.87 (0.74–0.94)	(− 0.18–0.12)	0.87 (0.72–0.94)
Cadence	0.83 (0.66–0.92)	(− 8.74–6.11)	0.75 (0.35–0.90)	(− 9.30–3.67)	0.74 (0.52–0.87)	(− 10.92–7.92)	0.77 (0.62–0.88)
Stride length	0.97 (0.91–0.99)	(− 0.09–0.05)	0.95 (0.58–0.98)	(− 0.10–0.02)	0.97 (0.91–0.98)	(− 0.09–0.05)	0.96 (0.89–0.98)
DS	0.88 (0.70–0.95)	(− 2.11–4.16)	0.93 (0.82–0.97)	(− 1.86–3.37)	0.96 (0.92–0.98)	(− 2.35–1.81)	0.92 (0.85–0.96)
Swing	0.75 (0.53–0.88)	(− 3.01–2.76)	0.90 (0.79–0.95)	(− 2.02–1.37)	0.69 (0.44–0.84)	(− 3.53–3.13)	0.78 (0.64–0.88)
Stance	0.75 (0.53–0.88)	(− 2.76–3.01)	0.90 (0.79–0.95)	(− 1.37–2.02)	0.69 (0.44–0.84)	(− 3.13–3.53)	0.78 (0.64–0.88)
LDr	0.97 (0.94–0.99)	(− 1.48–1.26)	0.94 (0.85–0.97)	(− 2.32–1.28)	0.95 (0.89–0.98)	(− 1.89–1.07)	0.95 (0.91–0.98)
FFr	0.87 (0.75–0.94)	(− 4.54–5.1)	0.92 (0.78–0.97)	(− 2.09–4.18)	0.84 (0.69–0.92)	(− 4.55–6.08)	0.88 (0.79–0.94)
Pur	0.87 (0.74–0.94)	(− 4.58–4.24)	0.94 (0.87–0.97)	(− 2.97–1.92)	0.88 (0.76–0.94)	(− 4.67–3.95)	0.89 (0.81–0.94)
*Normalized spatiotemporal gait parameters*							
Speed N	0.91 (0.73–0.96)	(− 0.05–0.02)	0.83 (0.29–0.94)	(− 0.06–0.02)	0.87 (0.73–0.94)	(− 0.06–0.04)	0.87 (0.71–0.94)
Cadence N	0.75 (0.52–0.87)	(− 2.73–1.92)	0.64 (0.20–0.84)	(− 2.86–1.12)	0.63 (0.35–0.81)	(− 3.41–2.48)	0.67 (0.48–0.82)
Slength N	0.95 (0.87–0.98)	(− 0.09–0.05)	0.93 (0.46–0.98)	(− 0.10–0.02)	0.95 (0.88–0.98)	(− 0.09–0.05)	0.94 (0.85–0.97)
*Symmetry index gait parameters*							
Speed SI	0.11 (− 0.24–0.45)	(− 7.13–5.15)	0.20 (− 0.12–0.51)	(− 6.24–3.74)	0.07 (− 0.31–0.42)	(− 7.78–7.27)	0.11 (− 0.09–0.37)
Cadence SI	0.00 (− 0.37–0.37)	(− 6.36–5.26)	0.16 (− 0.22–0.5)	(− 4.10- 3.49)	0.02 (− 0.35–0.38)	(− 6.70–7.18)	0.03 (− 0.16–0.28)
Slength SI	0.49 (0.15–0.72)	(− 4.06–3.43)	0.52 (0.20- 0.74)	(− 3.76–2.88)	0.37 (0.01–0.65)	(− 4.43–4.18)	0.45 (0.23–0.67)
Swing SI	0.49 (0.15–0.73)	(− 5.51–6.5)	0.56 (0.25–0.77)	(− 6.27–5.86)	0.18 (− 0.20–0.51)	(− 8.37–6.97)	0.42 (0.19–0.64)
Stance SI	0.48 (0.14–0.72)	(− 3.69–4.34)	0.58 (0.27–0.78)	(− 4.09–3.77)	0.23 (− (− 0.15–0.55)	(− 5.29–4.33)	0.43 (0.21–0.65)
LDr SI	0.50 (0.17–0.73)	(− 18.84–14.14)	0.38 (0.02–0.66)	(− 20.98–19.54)	0.54 (0.21–0.75)	(− 17.04–20.3)	0.47 (0.25–0.68)
FFr SI	0.48 (0.15–0.72)	(− 9.29–7.01)	0.67 (0.41–0.83)	(− 6.08–4.82)	0.24 (− 0.14–0.56)	(− 10.40–11.42)	0.43 (0.20- 0.65)
Pur SI	0.15 (− 0.22–0.49)	(− 12.62–9.95)	0.36 (0.01–0.64)	(− 11.22–8.41)	0.27 (− 0.10–0.58)	(− 12.66–12.53)	0.26 (0.04–0.51)
*Symmetry ratio gait parameters*							
Speed SR	0.00 (− 0.33–0.35)	(− 0.09–0.06)	0.23 (− 0.12–0.54)	(− 0.07–0.05)	0.17 (− 0.21–0.51)	(− 0.08–0.08)	0.10 (− 0.10–0.36)
Cadence SR	0.20 (− 0.16–0.52)	(− 0.07–0.05)	0.01 (− 0.34–0.37)	(− 0.06–0.04)	0.09 (− 0.28–0.45)	(− 0.08–0.08)	0.11 (− 0.10–0.37)
Slength SR	0.00 (− 0.37–0.37)	(− 0.07–0.06)	0.50 (0.17–0.73)	(− 0.05–0.03)	0.35 (− 0.02–0.63)	(− 0.05–0.05)	0.26 (0.03–0.51)
Swing SR	0.57 (0.26–0.77)	(− 0.09–0.05)	0.66 (0.34–0.83)	(− 0.09–0.05)	0.59 (0.29–0.79)	(− 0.09–0.08)	0.61 (0.41–0.78)
Stance SR	0.58 (0.27–0.78)	(− 0.04–0.06)	0.71 (0.40–0.86)	(− 0.03–0.05)	0.63 (0.35–0.81)	(− 0.05–0.05)	0.64 (0.45–0.80)
LDr SR	0.82 (0.64–0.91)	(− 0.20–0.18)	0.59 (0.29–0.79)	(− 0.29–0.30)	0.71 (0.47–0.86)	(− 0.25–0.28)	0.70 (0.53–0.84)
FFr SR	0.77 (0.56–0.88)	(− 0.08–0.08)	0.47 (0.13–0.71)	(− 0.11–0.11)	0.57 (0.25–0.77)	(− 0.12–0.11)	0.61 (0.40–0.78)
Pur SR	0.56 (0.25–0.77)	(− 0.15–0.14)	0.56 (0.24–0.77)	(− 0.15–0.14)	0.62 (0.33–0.8)	(− 0.15–0.16)	0.58 (0.37–0.76)

Abbreviations: ICC = intraclass correlation coefficient, LOA = limits of agreement, SI = symmetry index, SR = symmetry ratio, lb = lower bound, ub = upper bound, DS = double support, LDr = Load Ratio, FFr = Foot flat ratio, Pur = Push ratio, N = normalized.

**Table 3:** Intraclass correlation coefficients (ICC) and limits of agreement (LOA) values for spatiotemporal gait parameters obtained in dual task condition between the mean of the first and second trials (T1-T2), the first and the third trials (T1-T3), the second and the third trials (T2-T3) and the means of the three consecutive trials (T1-T2-T3).

	ICC 12 (lb–ub)	LOA (lb–ub)	ICC 13 (lb–ub)	LOA (lb–ub)	ICC 23 (lb–ub)	LOA (lb–ub)	ICC(123)
**DUAL TASK**							
*Spatiotemporal gait parameters*							
Speed	0.84 (0.64–0.92)	(− 0.20–0.11)	0.83 (0.63–0.92)	(− 0.19–0.1)	0.94 (0.87–0.97)	(− 0.11–0.11)	0.87 (0.77–0.94)
Cadence	0.83 (0.66–0.92)	(− 6.36–4.29)	0.78 (0.58–0.89)	(− 7.80–5.67)	0.90 (0.79–0.95)	(− 4.78–4.72)	0.83 (0.72–0.91)
Stride length	0.92 (0.79–0.96)	(− 0.14–0.07)	0.93 (0.81–0.97)	(− 0.12–0.06)	0.97 (0.93–0.98)	(− 0.07–0.08)	0.94 (0.88–0.97)
DS	0.90 (0.74–0.96)	(− 1.90–3.75)	0.82 (0.62–0.91)	(− 3.11–5.16)	0.95 (0.89–0.98)	(− 2.27–2.48)	0.89 (0.79–0.94)
Swing	0.85 (0.69–0.93)	(− 2.42–1.5)	0.76 (0.55–0.88)	(− 3.05–2.06)	0.90 (0.79–0.95)	(− 1.80–1.72)	0.84 (0.72–0.91)
Stance	0.85 (0.69–0.93)	(− 1.50–2.42)	0.76 (0.55–0.88)	(− 2.06–3.05)	0.90 (0.79–0.95)	(− 1.72–1.8)	0.84 (0.72–0.91)
LDr	0.94 (0.89–0.97)	(− 1.81–1.63)	0.93 (0.85–0.96)	(− 2.28–1.75)	0.97 (0.94–0.99)	(− 1.47–1.12)	0.95 (0.91–0.97)
FFr	0.86 (0.72–0.93)	(− 4.87–5.12)	0.88 (0.75–0.94)	(− 4.10–5.12)	0.96 (0.92–0.98)	(− 2.20- 2.97)	0.90 (0.83–0.95)
Pur	0.87 (0.75–0.94)	(− 4.11–4.04)	0.89 (0.78–0.95)	(− 4.07–3.58)	0.96 (0.93–0.98)	(− 2.52–2.1)	0.91 (0.85–0.96)
*Normalized spatiotemporal gait parameters*							
Speed N	0.78 (0.54–0.90)	(− 0.07–0.04)	0.78 (0.53–0.89)	(− 0.06–0.03)	0.92 (0.83–0.96)	(− 0.04–0.04)	0.83 (0.70–0.91)
Cadence N	0.84 (0.68–0.92)	(− 1.96–1.31)	0.76 (0.55–0.88)	(− 2.38–1.74)	0.89 (0.78–0.95)	(− 1.46–1.46)	0.83 (0.71–0.91)
Slength N	0.81 (0.58–0.91)	(− 0.15–0.08)	0.84 (0.60–0.93)	(− 0.12–0.06)	0.93 (0.85–0.96)	(− 0.08–0.08)	0.86 (0.74–0.93)
*Symmetry index gait parameters*							
Speed SI	0.37 (0.01–0.65)	(− 6.37–6.69)	0.08 (− 0.30–0.43)	(− 7.33–8.3)	0.23 (− 0.14–0.55)	(− 6.92–7.57)	0.22 (0–0.48)
Cadence SI	0.08 (− 0.29–0.44)	(− 5.79–6.02)	0.06 (− 0.31–0.42)	(− 8.31–7.9)	0.00 (− 0.37–0.37)	(− 7.38–6.74)	0.03 (− 0.16–0.28)
Slength SI	0.34 (− 0.02–0.63)	(− 5.20- 5.8)	0.25 (− 0.13–0.56)	(− 5.74–6.36)	0.35 (− 0.01–0.64)	(− 5.56–5.58)	0.31 (0.08–0.56)
Swing SI	0.75 (0.54–0.88)	(− 4.64–5.1)	0.52 (0.20–0.75)	(− 7.73–7.54)	0.67 (0.40- 0.83)	(− 6.51–5.86)	0.64 (0.45–0.8)
Stance SI	0.81 (0.64–0.91)	(− 2.82–3.02)	0.60 (0.30–0.79)	(− 4.72–4.56)	0.74 (0.51–0.87)	(− 3.86–3.51)	0.71 (0.54–0.84)
LDr SI	0.68 (0.42–0.84)	(− 14.42–13.66)	0.72 (0.48–0.86)	(− 13.33–12.47)	0.58 (0.27–0.78)	(− 15.56–15.47)	0.66 (0.47–0.81)
FFr SI	0.49 (0.15–0.73)	(− 7.79–8.44)	0.52 (0.19–0.74)	(− 9.91–9.02)	0.54 (0.22–0.76)	(− 9.28–7.75)	0.52 (0.30–0.71)
Pur SI	0.88 (0.75–0.94)	(− 6.94–7.81)	0.66 (0.39–0.83)	(− 13.09–12.49)	0.73 (0.50–0.86)	(− 11.02–9.55)	0.75 (0.60–0.87)
*Symmetry ratio gait parameters*							
Speed SR	0.10 (− 0.24–0.44)	(− 0.08–0.11)	0.08 (− 0.30–0.43)	(− 0.08–0.09)	0.28 (− 0.08–0.58)	(− 0.10–0.07)	0.16 (− 0.05–0.42)
Cadence SR	0.25 (− 0.10–0.56)	(− 0.05–0.07)	0.00 (− 0.37–0.37)	(− 0.10–0.10)	0.17 (− 0.20- 0.51)	(− 0.08–0.07)	0.10 (− 0.10–0.36)
Slength SR	0.18 (− 0.16–0.5)	(− 0.06–0.09)	0.24 (− 0.14–0.56)	(− 0.06–0.07)	0.32 (− 0.03–0.61)	(− 0.08–0.06)	0.24 (0.03–0.49)
Swing SR	0.64 (0.36–0.82)	(− 0.07–0.09)	0.57 (0.26–0.77)	(− 0.11–0.10)	0.54 (0.22–0.76)	(− 0.11–0.09)	0.58 (0.37–0.76)
Stance SR	0.74 (0.52–0.87)	(− 0.05–0.04)	0.68 (0.42–0.84)	(− 0.06–0.06)	0.67 (0.40- 0.83)	(− 0.05–0.06)	0.69 (0.51–0.83)
LDr SR	0.75 (0.52–0.87)	(− 0.21–0.24)	0.73 (0.50–0.87)	(− 0.23–0.22)	0.74 (0.51–0.87)	(− 0.24–0.20)	0.74 (0.58–0.86)
FFr SR	0.45 (0.10–0.70)	(− 0.12–0.09)	0.42 (0.06–0.68)	(− 0.13–0.12)	0.64 (0.36–0.81)	(− 0.09–0.11)	0.50 (0.28–0.71)
Pur SR	0.82 (0.65–0.91)	(− 0.11–0.15)	0.74 (0.52–0.87)	(− 0.16–0.17)	0.83 (0.67–0.92)	(− 0.14–0.11)	0.80 (0.66–0.89)

Abbreviations: ICC = intraclass correlation coefficient, LOA = limits of agreement, SI = symmetry index, SR = symmetry ratio, lb = lower bound, ub = upper bound, DS = double support, LDr = Load Ratio, FFr = Foot flat ratio, Pur = Push ratio, N = normalized.

On the one hand, results showed that almost all ICC of spatiotemporal gait parameters were above 0.81 and considered as almost perfect relative reliability in single- and dual-task conditions^49^, except for cadence, normalized cadence, swing, and stance with ICC above 0.61 and considered as substantial reliability. On the other hand, symmetry index (SI) and symmetry ratio (SR) gait parameters showed lower ICC values with ICC ranging from 0 to 0.25 for speed and cadence, and from 0.15 to 0.88 for slength, swing, stance, LDr, FFr and PUr.

**Figure 1:**
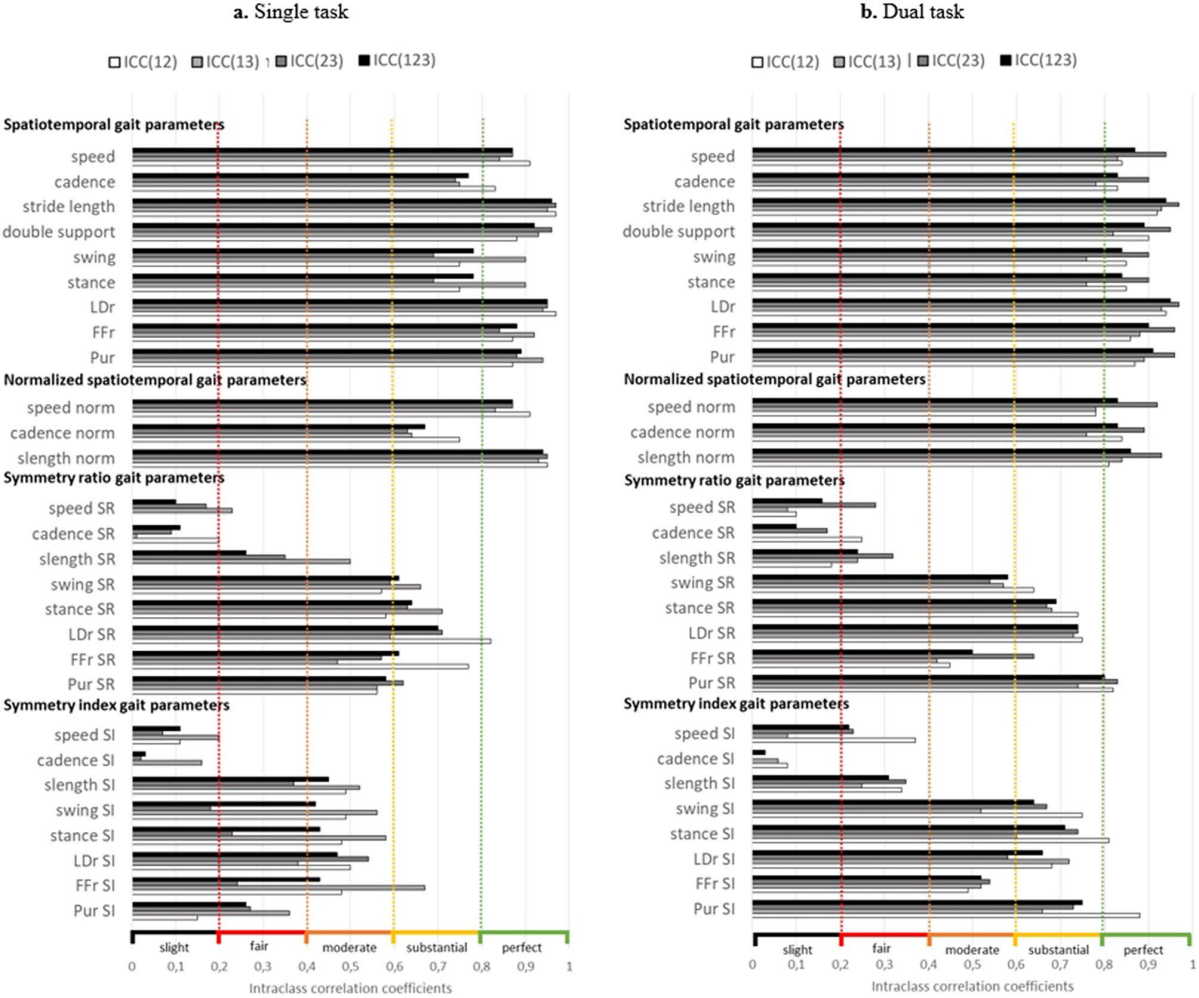
Intraclass correlation coefficients calculated with the means of trial 1–2, 1–3, 2–3 and 1–2-3 in single-task (**a**) and dual-task (**b**) conditions; *Dotted color lines correspond to relative reliability thresholds with: slight (black: 0.00* < *ICC* < *0.2), fair (red: 0.21* < *ICC* < *0.4), moderate (orange: 0.41* < *ICC* < *0.6), substantial (yellow: 0.61* < *ICC* < *0.8), and almost perfect (green: 0.81* < *ICC* < *1.0) reliability* ^49^. Abbreviations: LDr = Load Ratio, FFr = Foot Flat ratio, PUr = Push ratio, nprm = normalized, SI = symmetry index, SR = symmetry ratio, slength = stride length

Figure 1 present relative reliability with ICC values for spatiotemporal and symmetry gait parameters in the single-task and the dual-task conditions.

Table 4 (single task condition) and Table 5 (dual task condition) report absolute reliability with standard error of measurement (SEM) and minimal detectable change (MDC) in the same unit as the parameter and in percentage, calculated between each trial (T1-T2, T1-T3, T2-T3 and T1-T2-T3). The results showed that SEM percentages were comprised between 1.05 and 6.58% in the single-task condition for spatiotemporal and normalized spatiotemporal gait parameters. For symmetry ratio, SEM percentage ranged from 1.66 to 27.22%, while symmetry index ranged from 42.76 to 193.73%. In the dual-task condition, comparable values for SEM and MDC were found.

**Table 4:** Standard error of measurement (SEM) and minimal detectable change (MDC) for trial 1 and 2, 1 and 3, 2 and 3 and 1, 2 and 3 in single task condition.

	Trial 1 & 2				Trial 1 & 3				Trial 2 & 3				Trial 1 & 2 & 3			
	SEM	MDC	SEM (%)	MDC (%)	SEM	MDC	SEM (%)	MDC (%)	SEM	MDC	SEM (%)	MDC (%)	SEM	MDC	SEM (%)	MDC (%)
**SINGLE TASK**																
*Spatiotemporal gait parameters*																
Speed	0.05	0.13	3.18	8.82	0.06	0.18	4.37	12.11	0.06	0.16	3.79	10.5	0.06	0.16	3.82	10.59
Cadence	2.73	7.58	2.41	6.68	2.92	8.10	2.56	7.09	3.43	9.52	2.99	8.29	3.05	8.44	2.67	7.39
Stride length	0.03	0.07	1.75	4.84	0.03	0.09	2.18	6.04	0.03	0.07	1.74	4.82	0.03	0.08	1.89	5.25
DS	1.29	3.58	6.58	18.25	1.05	2.9	5.30	14.69	0.75	2.07	3.87	10.73	1.05	2.91	5.38	14.9
Swing	1.01	2.79	2.53	7.02	0.63	1.75	1.58	4.38	1.17	3.24	2.93	8.11	0.96	2.66	2.41	6.67
Stance	1.01	2.79	1.67	4.64	0.63	1.75	1.05	2.91	1.17	3.24	1.95	5.40	0.96	2.66	1.60	4.42
LDr	0.48	1.33	3.87	10.74	0.72	2.00	5.70	15.8	0.59	1.63	4.63	12.82	0.60	1.67	4.79	13.27
FFr	1.69	4.68	3.24	8.99	1.31	3.62	2.53	7.01	1.92	5.33	3.74	10.36	1.65	4.58	3.20	8.86
Pur	1.54	4.26	4.32	11.99	0.92	2.56	2.58	7.16	1.52	4.21	4.24	11.75	1.35	3.75	3.79	10.49
*Normalized spatiotemporal gait parameters*																
Speed N	0.02	0.04	3.17	8.79	0.02	0.06	4.36	12.08	0.02	0.05	3.75	10.38	0.02	0.05	3.80	10.53
Cadence N	0.85	2.37	2.44	6.76	0.89	2.47	2.53	7.00	1.07	2.98	3.03	8.4	0.95	2.62	2.69	7.45
Slength N	0.03	0.08	1.75	4.85	0.04	0.10	2.16	6.00	0.03	0.08	1.70	4.72	0.03	0.09	1.88	5.21
*Symmetry index gait parameters*																
Speed SI	2.24	6.22	97.51	270.29	1.90	5.26	78.09	216.46	2.65	7.35	90.64	251.25	2.30	6.37	90.06	249.63
Cadence SI	2.07	5.73	186.3	516.4	1.35	3.74	136.8	379.18	2.45	6.78	193.73	536.98	2.01	5.57	179.59	497.79
Slength SI	1.33	3.68	55.34	153.41	1.20	3.32	48.68	134.94	1.51	4.19	57.71	159.97	1.35	3.74	54.14	150.08
Swing SI	2.13	5.9	63.92	177.17	2.12	5.88	57.71	159.97	2.74	7.58	79.74	221.04	2.34	6.49	67.29	186.51
Stance SI	1.42	3.94	64.96	180.06	1.38	3.82	56.65	157.04	1.72	4.77	75.86	210.27	1.51	4.19	65.8	182.39
LDr SI	5.97	16.56	46.13	127.86	7.11	19.7	58.58	162.38	6.62	18.35	49.73	137.86	6.58	18.23	51.39	142.44
FFr SI	2.95	8.17	61.25	169.77	1.95	5.4	42.76	118.53	3.84	10.65	74.91	207.64	3.01	8.34	62.27	172.6
Pur SI	4.07	11.28	65.52	181.60	3.56	9.86	57.00	157.99	4.41	12.23	63.83	176.94	4.03	11.17	62.42	173.03
*Symmetry ratio gait parameters*																
Speed SR	0.03	0.07	2.64	7.32	0.02	0.06	2.27	6.3	0.03	0.08	2.80	7.75	0.03	0.07	2.64	7.31
Cadence SR	0.02	0.06	2.23	6.19	0.02	0.05	1.83	5.08	0.03	0.08	2.78	7.70	0.02	0.06	2.32	6.44
Slength SR	0.02	0.06	2.09	5.8	0.01	0.04	1.42	3.93	0.02	0.05	1.66	4.60	0.02	0.05	1.76	4.89
Swing SR	0.03	0.07	2.73	7.57	0.03	0.08	2.75	7.61	0.03	0.08	2.93	8.12	0.03	0.08	2.81	7.79
Stance SR	0.02	0.05	1.79	4.95	0.02	0.05	1.67	4.64	0.02	0.05	1.81	5.00	0.02	0.05	1.76	4.88
LDr SR	0.07	0.19	6.37	17.64	0.10	0.28	9.82	27.22	0.09	0.26	8.88	24.62	0.09	0.25	8.45	23.41
FFr SR	0.03	0.08	2.85	7.91	0.04	0.11	3.93	10.89	0.04	0.11	4.21	11.67	0.04	0.10	3.70	10.25
Pur SR	0.05	0.14	4.93	13.67	0.05	0.14	4.97	13.77	0.05	0.15	5.28	14.63	0.05	0.14	5.05	14.00

Abbreviations: SEM = standard error of measurement, MDC = Minimum detectable change, DS = double support, LDr = Load Ratio, FFr = Foot flat ratio, Pur = Push ratio, SI = symmetry index, SR = symmetry ratio, N = normalized.

**Table 5:** Standard error of measurement (SEM) and minimal detectable change (MDC) for trial 1 and 2, 1 and 3, 2 and 3 and 1, 2 and 3 in dual task condition.

	Trial 1 & 2				Trial 1 & 3				Trial 2 & 3				Trial 1 & 2 & 3			
	SEM	MDC	SEM (%)	MDC (%)	SEM	MDC	SEM (%)	MDC (%)	SEM	MDC	SEM (%)	MDC (%)	SEM	MDC	SEM (%)	MDC (%)
**DUAL TASK**																
*Spatiotemporal gait parameters*																
Speed	0.06	0.17	4.31	11.95	0.06	0.16	4.09	11.33	0.04	0.11	2.66	7.37	0.05	0.15	3.75	10.39
Cadence	1.98	5.49	1.74	4.81	2.45	6.80	2.15	5.96	1.65	4.58	1.44	3.99	2.05	5.68	1.79	4.97
Stride length	0.04	0.12	2.90	8.04	0.04	0.10	2.45	6.8	0.03	0.07	1.80	4.98	0.04	0.10	2.42	6.70
DS	1.17	3.23	5.79	16.05	1.60	4.43	7.95	22.03	0.83	2.30	4.22	11.7	1.24	3.43	6.20	17.17
Swing	0.75	2.08	1.89	5.24	0.95	2.63	2.4	6.64	0.61	1.70	1.54	4.26	0.78	2.17	1.97	5.46
Stance	0.75	2.08	1.24	3.44	0.95	2.63	1.57	4.36	0.61	1.70	1.02	2.82	0.78	2.17	1.30	3.59
LDr	0.60	1.67	4.66	12.93	0.72	2.00	5.57	15.43	0.47	1.29	3.57	9.91	0.6	1.67	4.65	12.9
FFr	1.74	4.82	3.39	9.39	1.64	4.55	3.21	8.9	0.94	2.60	1.83	5.08	1.48	4.09	2.89	8.00
Pur	1.42	3.93	3.96	10.98	1.34	3.72	3.74	10.36	0.81	2.26	2.27	6.29	1.22	3.37	3.39	9.40
*Normalized spatiotemporal gait parameters*																
Speed N	0.02	0.06	4.29	11.88	0.02	0.05	4.12	11.41	0.01	0.04	2.67	7.41	0.02	0.05	3.76	10.41
Cadence N	0.61	1.69	1.73	4.80	0.75	2.08	2.13	5.90	0.51	1.41	1.44	3.99	0.63	1.74	1.78	4.95
Slength N	0.04	0.12	2.85	7.89	0.04	0.11	2.46	6.82	0.03	0.08	1.79	4.97	0.04	0.11	2.40	6.67
*Symmetry index gait parameters*																
Speed SI	2.29	6.34	64.65	179.2	2.77	7.67	81.88	226.97	2.55	7.07	77.31	214.3	2.54	7.04	74.58	206.71
Cadence SI	2.08	5.75	154.87	429.29	2.85	7.90	190.13	527.01	2.43	6.74	168.72	467.66	2.49	6.91	174.74	484.36
Slength SI	1.94	5.37	53.57	148.49	2.13	5.91	59.04	163.64	1.95	5.40	56.31	156.08	2.01	5.56	56.29	156.02
Swing SI	1.70	4.72	41.56	115.18	2.67	7.40	62.56	173.4	2.17	6.01	52.24	144.79	2.21	6.12	52.94	146.73
Stance SI	1.02	2.82	37.84	104.88	1.62	4.49	58.36	161.77	1.29	3.57	47.26	130.99	1.33	3.68	48.61	134.74
LDr SI	4.90	13.59	42.49	117.79	4.50	12.49	38.97	108.03	5.41	15.01	46.09	127.76	4.94	13.69	42.54	117.91
FFr SI	2.85	7.89	56.62	156.93	3.32	9.20	61.38	170.14	3.02	8.37	57.55	159.53	3.06	8.49	58.57	162.34
Pur SI	2.58	7.16	32.61	90.39	4.46	12.37	53.87	149.33	3.62	10.03	44.87	124.36	3.62	10.05	44.8	124.17
*Symmetry ratio gait parameters*																
Speed SR	0.04	0.10	3.47	9.61	0.03	0.08	2.92	8.10	0.03	0.09	3.08	8.54	0.03	0.09	3.18	8.80
Cadence SR	0.02	0.06	2.30	6.39	0.04	0.10	3.50	9.70	0.03	0.08	2.72	7.54	0.03	0.08	2.91	8.06
Slength SR	0.03	0.08	2.82	7.80	0.02	0.06	2.17	6.03	0.03	0.07	2.56	7.11	0.03	0.07	2.54	7.04
Swing SR	0.03	0.08	3.03	8.39	0.04	0.10	3.72	10.31	0.04	0.10	3.63	10.07	0.03	0.09	3.47	9.61
Stance SR	0.02	0.05	1.75	4.84	0.02	0.06	2.12	5.87	0.02	0.06	2.07	5.74	0.02	0.06	1.98	5.48
LDr SR	0.08	0.22	7.64	21.19	0.08	0.22	7.58	21.02	0.08	0.22	7.61	21.11	0.08	0.22	7.59	21.04
FFr SR	0.04	0.11	4.01	11.12	0.04	0.12	4.50	12.48	0.03	0.10	3.60	9.99	0.04	0.11	4.05	11.22
Pur SR	0.05	0.13	4.38	12.15	0.06	0.16	5.44	15.07	0.04	0.12	4.25	11.78	0.05	0.14	4.71	13.05

Abbreviations: SEM = standard error of measurement, MDC = Minimum detectable change, DS = double support, LDr = Load Ratio, FFr = Foot flat ratio, Pur = Push ratio, SI = symmetry index, SR = symmetry ratio, N = normalized.

The comparisons of means of ICC between trials showed that slightly higher ICC and lower SEM and MDC were obtained when pooling gait parameters from the second and the third trials, compared to the first and second trials, to the first and third trials, or to the three trials in the single-task and the dual-task conditions.

**Figure 2:**
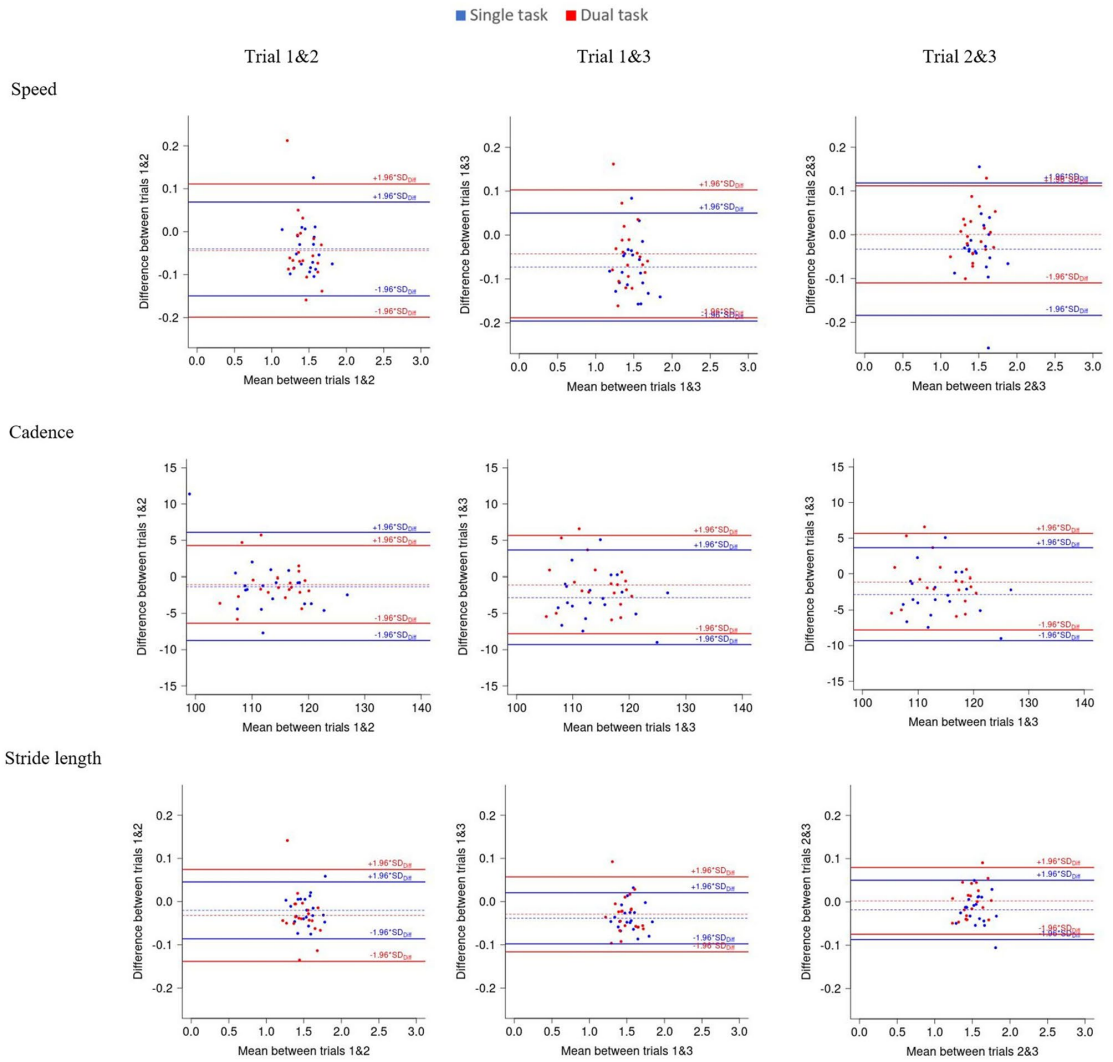
Bland and Altman plots for speed, stride length and cadence in single task (blue) and dual task (red) and for speed, stride length and cadence for trial 1 and 2, 1 and 3 and 2 and 3.

Bland and Altman plots for speed, cadence, and stride length in single task and dual task conditions can be found in Fig. 2.

### Intra-session relative and absolute reliability of dual-task effects on gait parameters

Table 6 presents relative reliability with ICC and LOA values on gait parameters for DTE and DTE%. Table 7 shows absolute reliability of DTE with SEM and minimal detectable change (MDC) calculated between each trial (T1-T2, T1-T3, T2-T3 and T1-T2-T3) for DTE and DTE% respectively. The results showed that DTE (0.00 < ICC < 0.82, 61.05 < SEM% < 3666.49) and DTE% (0.00 < ICC < 0.82, 68.03 < SEM% < 14,994.32) reliabilities were low. Relative reliability of DTE % expressed with ICC was comprised between 0.61 and 1.00 for speed, stride length and double support, between 0.21 to 0.41 for cadence, load ratio and foot flat ratio (0.21 < ICC < 0.41), and between 0.00 and 0.21 for swing, stance and push ratio.

**Table 6:** Intraclass correlation coefficients (ICC) and limits of agreement (LOA) values for dual task effects (DTE) and DTE percentage of gait parameters.

	ICC 12 (lb–ub)	LOA (lb–ub)	ICC 13 (lb–ub)	LOA (lb–ub)	ICC 23 (lb–ub)	LOA (lb–ub)	ICC(123)
**DTE for spatiotemporal gait parameters**							
Speed	0.84 (0.68–0.92)	(− 0.16–0.17)	0.77 (0.56–0.89)	(− 0.21–0.15)	0.70 (0.45–0.85)	(− 0.24–0.17)	0.77 (0.62–0.88)
Cadence	0.59 (0.29–0.79)	(− 9.49–8.93)	0.47 (0.15–0.71)	(− 10.58–7.07)	0.26 (− 0.11–0.57)	(− 13.32–10.37)	0.44 (0.21–0.66)
Stride length	0.81 (0.64–0.91)	(− 0.10–0.13)	0.82 (0.65–0.91)	(− 0.12–0.10)	0.80 (0.61–0.90)	(− 0.13–0.09)	0.81 (0.68–0.90)
DS	0.80 (0.62–0.90)	(− 2.80–3.00)	0.44 (0.09–0.69)	(− 4.69–4.15)	0.54 (0.23–0.76)	(− 4.15–3.41)	0.61 (0.41–0.78)
Swing	0.06 (− 0.32–0.42)	(− 3.60–4.26)	0.27 (− 0.10–0.58)	(− 2.92–3.26)	0.00 (− 0.37–0.37)	(− 4.40- 4.08)	0.08 (− 0.12–0.35)
Stance	0.06 (− 0.32–0.42)	(− 4.26–3.60)	0.27 (− 0.10–0.58)	(− 3.26–2.92)	0.00 (− 0.37–0.37)	(− 4.08–4.40)	0.08 (− 0.12–0.35)
LDr	0.55 (0.24–0.76)	(− 2.32–2.28)	0.44 (0.09–0.69)	(− 2.55–2.04)	0.60 (0.30–0.79)	(− 2.05–1.58)	0.53 (0.31–0.72)
FFr	0.19 (− 0.19–0.52)	(− 6.93–7.23)	0.45 (0.10–0.70)	(− 3.92–4.99)	0.36 (− 0.01–0.64)	(− 5.80–6.57)	0.31 (0.08–0.56)
Pur	0.19 (− 0.19–0.52)	(− 5.61–5.35)	0.44 (0.09–0.69)	(− 3.78–3.22)	0.27 (− 0.10–0.58)	(− 5.43–5.13)	0.27 (0.05–0.53)
**DTE% for spatiotemporal gait parameters**							
Speed	0.82 (0.64–0.91)	(− 11.84–11.96)	0.72 (0.48–0.86)	(− 15.34–11.16)	0.71 (0.47–0.85)	(− 14.97–10.68)	0.75 (0.60–0.87)
Cadence	0.50 (0.17–0.73)	(− 9.57–9.36)	0.44 (0.11–0.69)	(− 9.44–6.30)	0.18 (− 0.19–0.51)	(− 12.86–9.93)	0.37 (0.14–0.60)
Stride length	0.80 (0.61–0.90)	(− 7.50–8.82)	0.80 (0.62–0.90)	(− 8.16–7.01)	0.82 (0.65–0.91)	(− 8.08–5.60)	0.81 (0.68–0.90)
DS	0.80 (0.62–0.90)	(− 13.89–15.28)	0.46 (0.12–0.71)	(− 23.19–20.86)	0.58 (0.28–0.78)	(− 21.63–17.91)	0.62 (0.43–0.79)
Swing	0.02 (− 0.36–0.38)	(− 9.63–11.45)	0.26 (− 0.11–0.57)	(− 7.36–8.30)	0.00 (− 0.37–0.37)	(− 11.50–10.63)	0.06 (− 0.14–0.32)
Stance	0.08 (− 0.29–0.44)	(− 6.82–5.78)	0.28 (− 0.10–0.59)	(− 5.39–4.86)	0.00 (− 0.37–0.37)	(− 6.67–7.18)	0.10 (− 0.10–0.36)
LDr	0.60 (0.30–0.79)	(− 19.69–17.19)	0.50 (0.18–0.73)	(− 21.39–14.54)	0.61 (0.32–0.8)	(− 17.35 –13.00)	0.57 (0.36–0.75)
FFr	0.21 (− 0.16–0.54)	(− 12.57–13.37)	0.46 (0.11–0.71)	(− 7.69–9.65)	0.39 (0.03–0.66)	(− 10.84–12.01)	0.34 (0.11–0.58)
Pur	0.14 (− 0.24–0.48)	(− 16.91–16.98)	0.39 (0.03–0.66)	(− 10.76–8.97)	0.24 (− 0.14–0.56)	(− 16.92–15.06)	0.22 (0.00–0.48)

Abbreviations: DTE = dual task effects, DS = double support, LDr = Load Ratio, FFr = Foot flat ratio, Pur = Push ratio.

**Table 7:** Standard error of measurement (SEM) and minimal detectable change (MDC) for DTE and DTE percentage for trial 1–2-3.

	Trial 1 & 2				Trial 1 & 3				Trial 2 & 3				Trial 1 & 2 & 3			
	SEM	MDC	SEM (%)	MDC (%)	SEM	MDC	SEM (%)	MDC (%)	SEM	MDC	SEM (%)	MDC (%)	SEM	MDC	SEM (%)	MDC (%)
**DTE for spatiotemporal gait parameters**																
Speed	0.06	0.16	126.19	349.79	0.07	0.18	104.62	289.98	0.07	0.21	122.45	339.42	0.07	0.18	117.28	325.09
Cadence	3.22	8.92	498.11	1380.69	3.28	9.09	3662.49	10,151.91	4.27	11.84	1854.83	5141.32	3.62	10.05	3331.00	9233.07
Stride length	0.04	0.11	78.12	216.53	0.04	0.11	61.05	169.23	0.04	0.11	71.09	197.04	0.04	0.11	69.42	192.42
DS	1.01	2.81	197.19	546.58	1.56	4.31	475.75	1318.71	1.34	3.73	354.92	983.78	1.32	3.66	324.54	899.59
Swing	1.40	3.88	737.45	2044.12	1.09	3.02	404.00	1119.83	1.48	4.11	1419.36	3934.25	1.34	3.71	712.40	1974.67
Stance	1.40	3.88	737.45	2044.12	1.09	3.02	404.00	1119.83	1.48	4.11	1419.36	3934.25	1.34	3.71	712.40	1974.67
LDr	0.80	2.23	173.66	481.35	0.82	2.28	238.15	660.12	0.65	1.81	195.17	540.99	0.76	2.11	200.04	554.49
FFr	2.49	6.89	379.25	1051.24	1.60	4.44	344.80	955.72	2.18	6.05	563.33	1561.46	2.12	5.87	421.78	1169.10
Pur	1.92	5.33	998.55	2767.84	1.24	3.43	1039.32	2880.86	1.85	5.14	3540.55	9813.91	1.70	4.71	1398.41	3876.20
**DTE% for spatiotemporal gait parameters**																
Speed	4.14	11.48	158.63	439.71	4.83	13.38	131.03	363.19	4.70	13.02	128.48	356.12	4.56	12.63	137.4	380.85
Cadence	3.31	9.17	439.94	1219.46	2.93	8.12	14,994.32	41,562.14	4.12	11.41	12,533.95	34,742.33	3.49	9.67	1417.44	3928.95
Stride length	2.88	7.98	87.10	241.44	2.67	7.40	68.03	188.58	2.52	7.00	70.23	194.68	2.69	7.45	74.51	206.52
DS	5.10	14.13	149.98	415.71	7.74	21.46	313.57	869.18	7.02	19.46	249.26	690.93	6.69	18.55	231.15	640.73
Swing	3.76	10.43	936.00	2594.45	2.76	7.66	446.21	1236.84	3.84	10.64	2318.51	6426.59	3.51	9.74	887.62	2460.37
Stance	2.24	6.22	647.31	1794.26	1.81	5.00	380.79	1055.48	2.43	6.74	1135.14	3146.44	2.18	6.04	631.48	1750.38
LDr	6.49	18.00	136.88	379.40	6.65	18.44	181.93	504.28	5.49	15.23	181.26	502.42	6.24	17.28	163.65	453.62
FFr	4.56	12.63	410.23	1137.10	3.11	8.62	378.81	1050.01	4.02	11.14	647.06	1793.56	3.93	10.91	462.37	1281.63
Pur	5.95	16.49	752.32	2085.33	3.51	9.73	1078.75	2990.16	5.64	15.65	1646.36	4563.46	5.14	14.26	1057.93	2932.44

Abbreviations: DTE = dual task effects, SEM = standard error of measurement, MDC = minimum detectable change, DS = double support, LDr = Load Ratio, FFr = Foot flat ratio.

Bland and Altman plots for speed, cadence, and stride length DTE and DTE% can be found in Supplementary Fig. S2.

## Discussion

The aim of the present study was to assess the reliability of Physilog5 foot-worn IMUs to assess gait in healthy adults during the 10-m walk test in single and dual task conditions. The objectives were 1) to evaluate the reliability, standard error of measurement (SEM) and minimal detectable change (MDC) values of spatiotemporal gait parameters and symmetry gait parameters obtained, (2) to evaluate the reliability, SEM and MDC values of dual task effects obtained from gait parameters, and (3) to determine the number of trials required to ensure reliable gait assessment.

Relative reliability (ICC) was considered as substantial to perfect for spatiotemporal gait parameters in both single- and dual-task conditions. Speed and stride length were the most reliable parameters in line with previous studies on healthy participants^3^. In a study using also Physilog IMUs with patients after stroke, comparable results on relative reliability (0.639 < ICC0.999) were found^22^. Absolute reliability (SEM and MDC) is not always reported in reliability studies^3^ and SEM was reported by only one study using Physilog sensors in healthy participants for local dynamic stability parameter^54^. Absolute reliability on stroke patients was lower (i.e. higher SEM% = 3.4 < SEM% < 14.8) but could be explained by the typical pathologic gait associated with stroke and hemiparesis that could lead to more variability (illustrated with higher SEM on paretic leg)^22^. In a study on healthy adults (n = 39, mean age: 23.8 ± 6.2) assessing gait with foot-worn IMUs^15^, MDC in single task was comparable to the results of the present study with 0.12 m.s^−1^ for gait speed (between 0.13 and 0.16 m.s^−1^ in the present study), 0.11 for stride length (between 0.07 and 0.09 m in the present study). However, lower MDC were found for cadence (2.72^15^ vs between 7.58 and 9.52 steps/minute in the present study), and for stance and swing percentage (1.49^15^ vs between 1.75 and 3.29% in the present study). These differences could be explained by the algorithm used to assess gait parameters as one used Mobility Lab (APDM, Inc., Portland, OR) while the present study used Gait Analysis Software (V5.3.0) from Gait Up (CH). MDC is used to evaluate the difference to be considered “real” than measurement error. It is important to note that MDC values reported in the present study can be used for an individual subject to assess change in performance before and after intervention^15^. However, MDC used to determine meaningful improvement in a group of participants has to take into account the size of the group (n) and is calculated as: $MDC_{{group}} = MDC_{{individual}} \div \sqrt n$.

Foot-worn IMUs (Physilog5, BioAGM, Gait Up, CH) were thus reliable to assess spatiotemporal gait parameters during walking at comfortable speed, regardless of the single or dual-task condition in healthy adults. This was not the case for symmetry gait parameters which showed less reliability than spatiotemporal gait parameters. We believe that this result is of importance considering the popularity and the relevance of symmetry gait parameters in clinical setting^55^. Indeed, while under healthy conditions and unconstrained walking conditions, gait pattern is generally symmetric (i.e. the left and the right lower limbs behave similarly)^56^, increased levels of gait asymmetry are typically observed in a variety of clinical populations such as elderly fallers^57^, individuals with lower-limb amputations^58^, knee osteoarthritis^59^, hip arthroplasties^60^, or after knee anterior cruciate ligament reconstruction^61^, people with multiple sclerosis^62^, persons with diabetes and peripheral neuropathy^63^, patients with Lewy body disease^64^, with Parkinson disease^65,66^, or after a stroke^44,67^. For instance, Fling et al. 2018 recently reported that people with Parkinson disease exhibited significant increased spatial (e.g., step length) and temporal (e.g., step time) asymmetries of the lower extremities during gait than age-matched healthy control participants^66^. Recent results of Wei et al. (2017) further revealed that gait asymmetry is an important factor for the prediction of falls in stroke patients^68^. Also of note is that chronic gait asymmetry could also lead to increased risk of lower-limb overuse injuries and articular joint degeneration due to the increased weight bearing and propulsion demands placed on one lower limb during walking, in both pathological^69–72^ and healthy populations^73^. Along these lines, gait asymmetry can be used as (i) a metric to assess pathology status, patients’ functional recovery or disease progression, (ii) a target for interventions aiming to improve gait performance, and (iii) outcome to evaluate and compare different gait rehabilitation programs interventions^43,55,74–77^. Interestingly, various rehabilitation programs have been shown to improve gait to a more symmetric gait pattern in individuals with lower-limb amputations (see for review^78^), stroke survivors (see for review^79^) or patients with Parkinson disease^80,81^.

In the present study, two formulae widely employed in clinical practice, namely symmetry ratio and symmetry index, were used to quantify gait symmetry^9,43,82^. Relative and absolute reliabilities of symmetry index of spatiotemporal gait parameters were low, as indicated by low values of ICC, high values of SEM% and high values of MDC%. Accordingly, following existing recommendations^22,51,52^, we would not recommend using SI of spatiotemporal gait parameters for the experimental conditions used in the present study at least. Interestingly, the symmetry ratio (SR) of spatiotemporal gait parameters showed better relative reliability that SI, as indicated by higher values of ICC (except for speed and stride length) and better absolute reliability, as indicated by acceptable SEM% and MDC% (SEM% and MDC% < 20%). This result is of particular interest since SR is easier to interpret by clinicians than SI^43^. Indeed, a SR of 2 indicates that the right foot was twice faster than left foot^43^, while SI gives a percentage of asymmetry that is more difficult to interpret^9^. All in all, SR of spatiotemporal gait parameters should be preferred to SI by clinicians and researchers to assess gait symmetry.

Dual task effects are often used to assess the cost of performing another task while walking and permits comparisons between studies with various secondary task^26,45^. However, the reliability of DTE is not commonly assessed in gait reliability studies but has to be tested to allow the use of this indicator in gait studies^24^. In our study, DTE% showed slightly better reliability with higher ICC for almost all gait parameters. Relative reliability of DTE % was considered as substantial to almost perfect for speed, stride length and double support (0.61 < ICC < 1.00), fair to moderate for cadence, load ratio and foot flat ratio (0.21 < ICC < 0.41), and slight to fair for swing, stance and push ratio (0.00 < ICC < 0.21). SEM percentage were high for all DTE parameters (68.03 < SEM% < 14,994.32%). These results are in line with previous studies reporting DTE reliability (0.19 < ICC < 0.55^24^; 0.002 < ICC < 0.882^83^) in participants with (n = 23, mean age: 80.6 ± 6.4) and without (n = 27, mean age: 76.1 ± 5.6) cognitive impairment disorders using a counting secondary task. One explanation of lower reliability of DTE is that measurement errors of each single task and dual task conditions tends to inflation of the overall measurement error when combined to obtain the DTE^83^. Besides, we asked participants not to prioritize one task^26,37^ and some may have chosen a posture-first strategy^84^, while others may have chosen a posture-second strategy^85^, leading to variability of gait performance that could have been amplified with DTE calculation^24^. Besides, the reliability of gait performance in dual task conditions can be dependent of the complexity of the dual-task used as a concurrent task to gait^86,87^, of the level of cognitive state of participants^88^ or of the educational level for verbal fluency tasks^89^. Thus, researchers or clinicians should be aware of the poor reliability of DTE. Even if DTE% of spatiotemporal gait parameters showed slightly better reliability than DTE of spatiotemporal gait parameters, their relative and absolute reliabilities were low (low ICC, and high SEM% and MDC%). Following existing recommendations^22,51,52^, we would not recommend using DTE and DTE% of spatiotemporal gait parameters for the experimental conditions used in the present study at least.

In the present study, even if the reliability of spatiotemporal gait parameters was substantial to perfect in both the single-task and the dual-task conditions, we observed slightly better ICC (i.e. higher), SEM and MDC (i.e. lower) when the reliability was calculate from the second and the third trials for most of gait parameters. While numerous studies calculated gait parameters using the average of three trials in older adults^37,90^ or pathological populations^91^, the number of trials to ensure reliable measurements using intra-session reliability has been less investigated^92,93^. Our results showed that the first trial appears less reliable and are in line with those reported in previous studies on healthy^93^ and pathological populations^92^. Thus, the mean of at least two trials should be used for better reliability of spatiotemporal gait parameters and the first trial could be used, like other studies, as a warming trial^94^.

A first limitation of the study is the lack of formal cognitive assessment of the participants considering the presumable impact of individual cognitive function on gait performance^88^^95–99^. Growing evidence suggests that the reliance on cognitive control processes during walking can be increased when performing a more complex locomotor task^96^, or when concurrently executing a dual-task (see for reviews^88,98,99^). Furthermore, a recent systematic review concluded that individuals with cognitive impairment can demonstrate decrease of gait performance^98^. What is more, previous studies have reported that motor slowing preceded cognitive decline in healthy older adults, suggesting that a decrease of gait performance could be a predictor of cognitive decline (see for recent reviews^100,101^). Another limitation of the present study is that reliability was assessed during a single experimental session, which by definition did not permit the calculation of the inter-session reliability. Inter-session reliability refers to the extent of agreement between measurements of a measure in sessions performed at different moments (usually at 1 h or 1 week interval)^102^. It is used to describe error magnitude between two sessions of time to further study disease evolution or the impact of treatments^102,103^. Furthermore, reliability was tested in healthy middle-aged adults. To allow the use of these sensors in clinical practice, the reliability has to be further tested in elderly and pathological populations.

To conclude, foot-worn IMUs are reliable to assess spatiotemporal and symmetry ratio gait parameters in healthy participants in both single- and dual-task conditions. Reliability of symmetry index and DTE gait parameters was low and we do not recommend the use of these parameters for the experimental conditions used in the present study. Future studies should examine the number of strides necessary to ensure better reliability of these index to allow their use in clinical practice and research^104^. Future studies should also focus on the assessment of reliability of 10-m walk test in pathological populations and should open up perspectives with other clinical tests (e.g. 6MWT or Timed Up and Go test) that could capture different features of gait and mobility in daily life.

## Supplementary Information

Supplementary Information

## Data Availability

The data of the present manuscript can be available on demand to the corresponding author.
