# Supplementary material for: Spatio-temporal gait parameters obtained from foot-worn inertial sensors are reliable in healthy adults in single- and dual-task conditions
Source: Sci Rep. 2021 May 13;11:10229. doi: 10.1038/s41598-021-88794-4 (PMC8119721; doi:10.1038/s41598-021-88794-4)

Spatio-temporal gait parameters obtained from foot-worn inertial sensors are reliable in healthy adults in single- and dual-task conditions

J. Soulard[1,2]*, J. Vaillant[1], R. Balaguier[1], N. Vuillerme[1,3,4]

1. Univ. Grenoble Alpes, AGEIS, Grenoble, France
2. Grenoble Alpes University Hospital, Grenoble, France
3. LabCom Telecom4Health, University Grenoble Apes & Orange Labs, Grenoble, France
4. Institut Universitaire de France, Paris, France

Figure S1: Placement of foot-worn inertial Physilog sensors

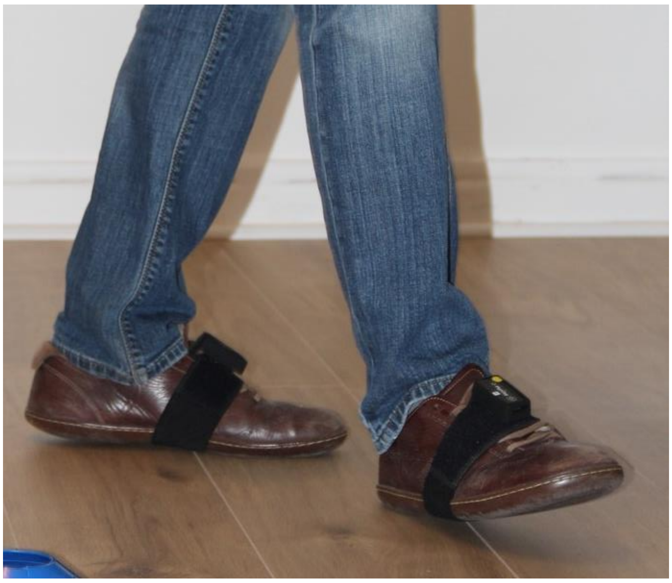

Figure S2: Bland and Altman plots for speed, stride length dual task effects (DTE) and DTE% for trial 1 and 2, 1 and 3 and 2 and 3

Speed DTE

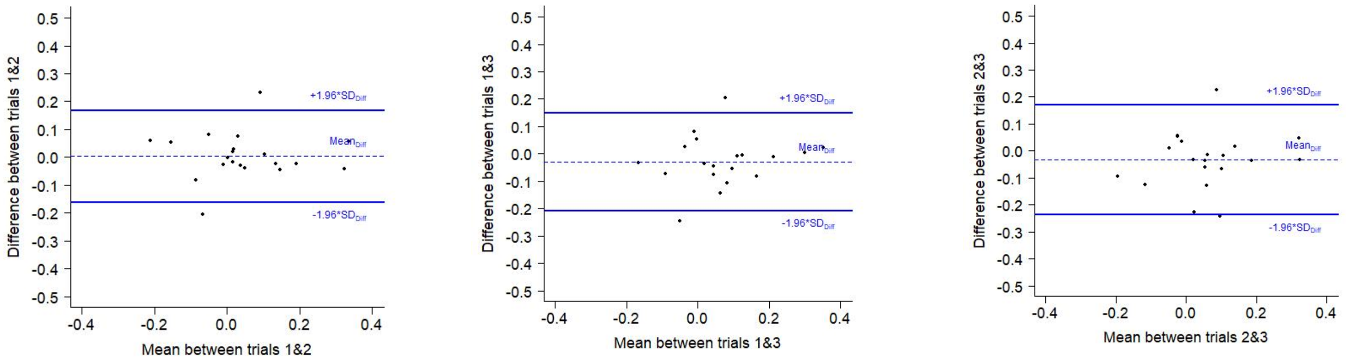

Cadence DTE

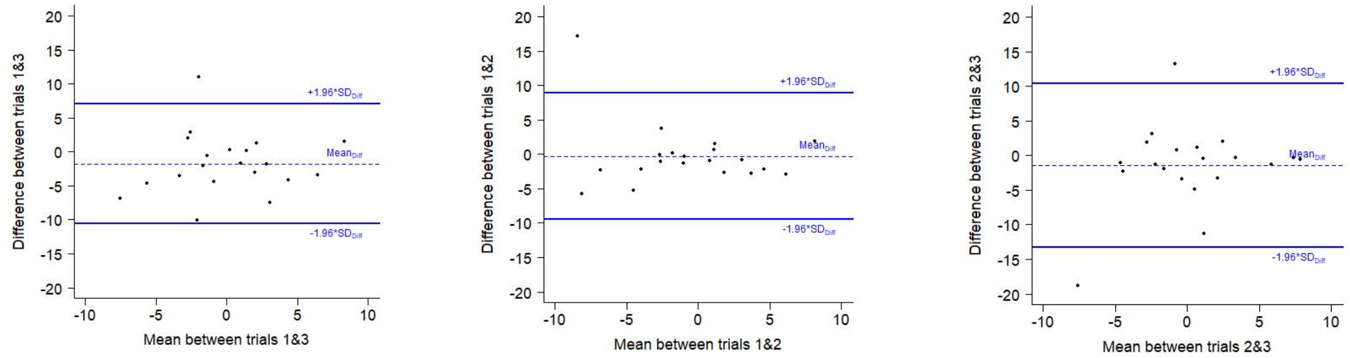

Stride length DTE

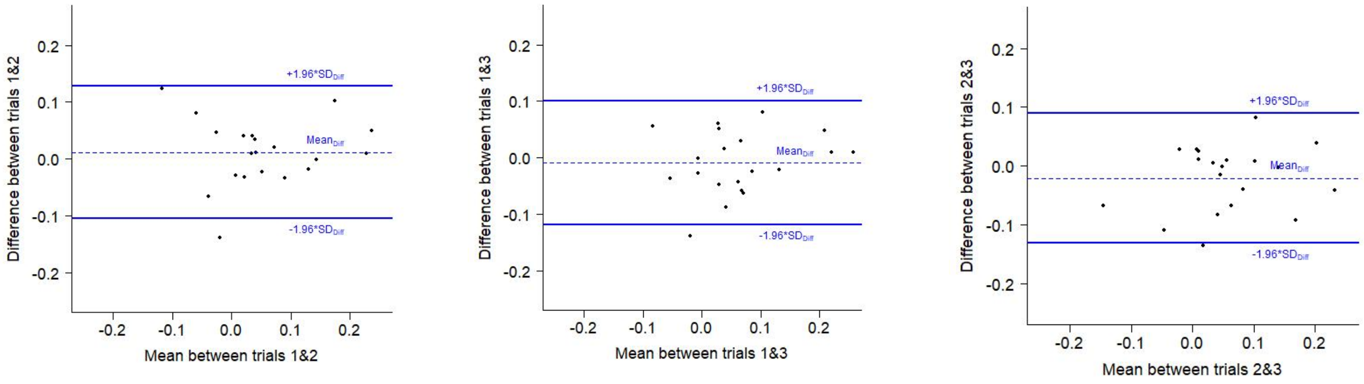

Speed DTE%

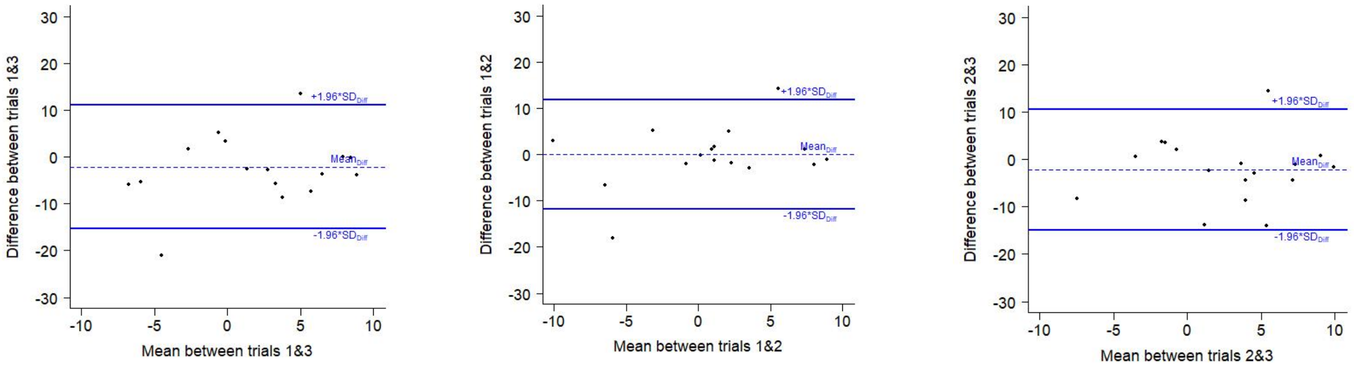

Cadence DTE%

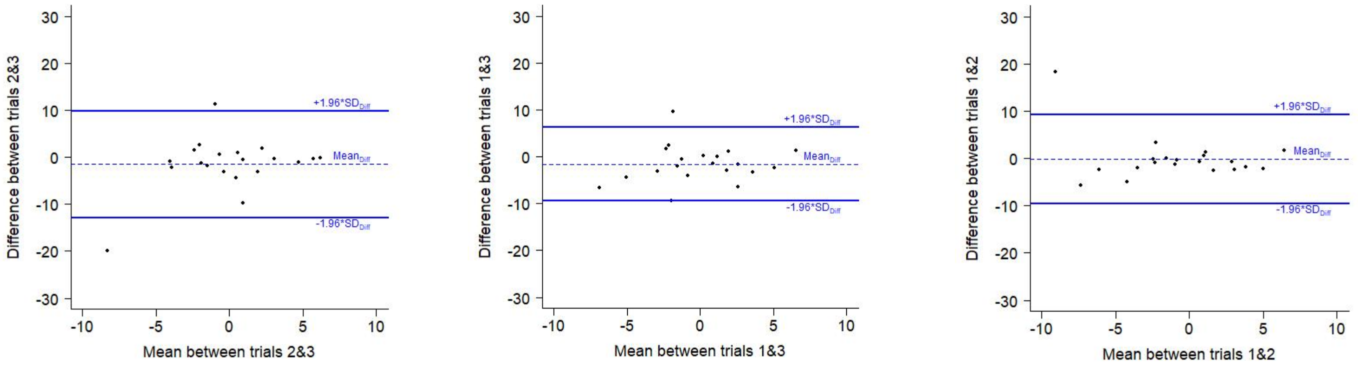

Stride length DTE%

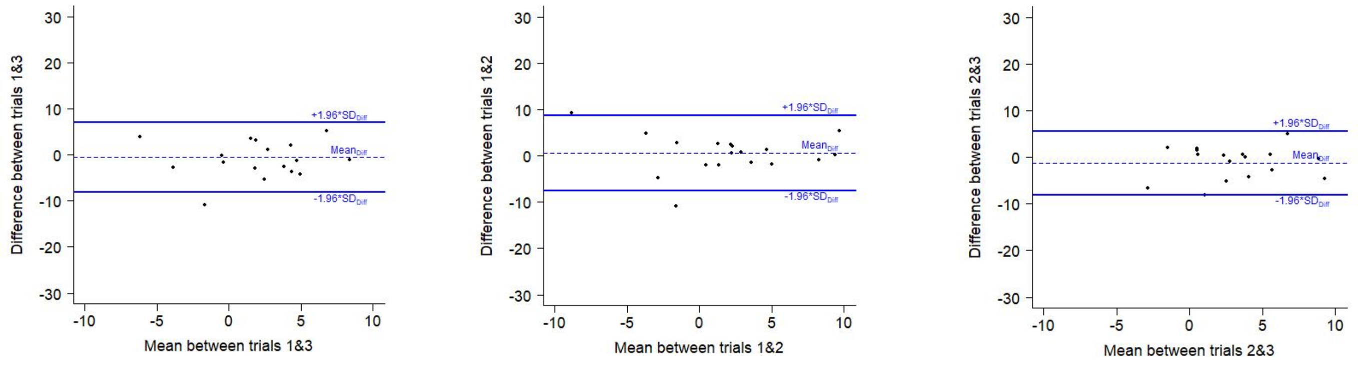

Supplement: Supplementary file 1 — Supplementary Information [file 41598_2021_88794_MOESM1_ESM.pdf]
